# Hidden Blood Loss and Its Possible Risk Factors in Full Endoscopic Lumbar Interbody Fusion

**DOI:** 10.3390/jpm13040674

**Published:** 2023-04-17

**Authors:** Zhilin Ge, Wenhua Zhao, Zhihua Wu, Jiahui He, Guangye Zhu, Zefeng Song, Jianchao Cui, Xiaobing Jiang, Weibo Yu

**Affiliations:** 1 First Clinical Medical College, Guangzhou University of Chinese Medicine, Guangzhou 510405, China; 2 Department of Spinal Surgery, The First Affiliated Hospital of Guangzhou University of Chinese Medicine, Guangzhou 510405, China; 3 Department of Orthopedics, The Third Affiliated Hospital of Southern Medical University, Guangzhou 510630, China

**Keywords:** endoscopy, spinal fusion, postoperative blood loss, risk factors

## Abstract

Background: Full endoscopic lumbar interbody fusion (Endo-LIF) is a representative recent emerging minimally invasive operation. The hidden blood loss (HBL) in an Endo-LIF procedure and its possible risk factors are still unclear. Methods: The blood loss (TBL) was calculated by Gross formula. Sex, age, BMI, hypertension, diabetes, ASA classification, fusion levels, surgical approach type, surgery time, preoperative RBC, HGB, Hct, PT, INR, APTT, Fg, postoperative mean arterial pressure, postoperative heart rate, Intraoperative blood loss (IBL), patient blood volume were included to investigate the possible risk factors by correlation analysis and multiple linear regression between variables and HBL. Results:Ninety-six patients (23 males, 73 females) who underwent Endo-LIF were retrospective analyzed in this study. The HBL was 240.11 (65.51, 460.31) mL (median [interquartile range]). Fusion levels (*p* = 0.002), age (*p* = 0.003), hypertension (*p* = 0.000), IBL (*p* = 0.012), PT (*p* = 0.016), preoperative HBG (*p* = 0.037) were the possible risk factors. Conclusion: Fusion levels, younger age, hypertension, prolonged PT, preoperative HBG are possible risk factors of HBL in an Endo-LIF procedure. More attention should be paid especially in multi-level minimally invasive surgery. The increase of fusion levels will lead to a considerable HBL.

## 1. Introduction

As an effective surgical treatment for lumbar spinal stenosis, lumbar interbody fusion has been widely used. The clinical efficacy of open surgery has been fully verified [1]. With the advancement of endoscopic technology and implants [2], as well as the exploration of its application [3,4], the full endoscopic lumbar interbody fusion (Endo-LIF) process has gradually been established [5,6,7,8]. With the purpose of reducing surgical trauma and accelerating recovery, the invasion of normal tissue and bleeding in open surgery [9] can be carefully controlled in an endoscopic approach. Since SEHAT et al. [10] proved the existence of perioperative hidden blood loss (HBL), this concept has been gradually accepted by surgeons. The amount of blood loss caused by infiltration in potential compartments or hemolysis is considerable [11]. Postoperative anemia is closely related to complications of spinal surgery, including rebleeding, wound healing disorder, motor deficit [12], etc. In addition, postoperative anemia may lead to a longer hospital stay and medical complications [13,14]. According to previous studies, the proportion of HBL in total blood loss in spinal surgery cannot be ignored [15,16,17]. For spinal surgeons, invisible HBL may lead to misestimation of blood loss, especially in minimally invasive surgery, which may hinder the management of anemia-related complications [18]. In the practice of full endoscopic interbody fusion surgery, we found that some patients without anemia preoperatively had a significant decrease in hemoglobin, even less than 80 g/L. We noticed that even with the smallest minimally invasive procedure, patients may still be associated with massive blood loss postoperatively. The hidden blood loss of Endo-LIF is seldom reported in present studies [19], and the risk factors are not clear. To provide advice on the prevention of complications corelated with blood loss, this study analyzed the hidden blood loss of Endo-LIF and its possible risk factors by retrospective analysis. So, we could estimate the amount of blood loss preoperatively in case of massive blood loss and get prepared in advance.

## 2. Materials and Methods

### 2.1. Patients

This was a retrospective clinical study. From September 2018 to June 2022, patients with lumbar degenerative disease in the First Affiliated Hospital of Guangzhou University of Traditional Chinese Medicine were analyzed. Inclusion criteria were: (1) Patients have the clear diagnosis of lumbar spinal stenosis or lumbar spondylolisthesis with surgical indications and were treated by Endo-LIF. (2) All operations were performed by one experienced operator and the same team of assistants. (3) Without history of lumbar trauma and surgery. Exclusion criteria: (1) Age less than 18 years; (2) Serious medical diseases or metabolic diseases, such as poorly controlled diabetes, uremia, etc. (3) With other non-degenerative spinal diseases, such as spinal tumors, spinal infections, ankylosing spondylitis, etc. (4) History of the abnormal coagulation function, such as cirrhosis, or use of anticoagulant or anti-platelet aggregation drugs; (5) preoperative anemia; (6) perioperative blood transfusion treatment.

Data of the patients were recorded, including sex, age, height, weight, BMI, hypertension, diabetes, ASA classification, fusion levels, surgical approach type, operative time, postoperative hospital stay, intraoperative blood loss (IBL), preoperative platelets (PLT) count, preoperative and postoperative red blood cell counting (RBC), Hemoglobin (HGB), hematocrit (Hct), and coagulation indicators, including: prothrombin time (PT), international normalized ratio(INR), activated partial thromboplastin time (APTT), fibrinogen (Fg), postoperative mean arterial pressure, postoperative heart rate. This study was approved by the Hospital Ethics Committee.

### 2.2. Surgical Procedures

All patients were placed in the prone position under general anesthesia on a fluoroscopic operating table. Based on the difference of surgical segment, etiology characteristics and estimation of decompression range, the surgical approach might be different. Generally, the trans-Kambin’s triangle approach was performed when the surgical segment was L4/5 or above, and extensive decompression range was required at the ventral of the nerve root. The interlaminar approach was applied at L5/S1 or when dorsal decompression of the spinal canal was required. Trans-Kambin approach: intervertebral space of surgical segments was located by fluoros [12] copy, a line 5–10° to the horizontal of the disc along the upper inner edge of the inferior pedicle was drawn. About 8–10 cm from the spinous process, a puncture to the foramen area was performed under the guidance of fluoroscopy. After foraminoplasty, the working cannula and endoscope were inroduced. Identifying the disc and looking for the rupture of the fibrous annulus, then the nucleus pulposus and cartilage endplate was removed. Withdraw from the disc after the endplate preparation completed. Identification of the nerve root and decompression was performed. The completion of decompression could be characterized by the pulsation of the nerve root and dural sac. After placing the guidewire into the disc space, the funnel was inserted along the guidewire for an allogeneic bone graft. The cage was implanted by a special nerve blocker and confirmed by fluoroscopy.

Interlaminar approach: the skin incision point was located under fluoroscopy at the intersection of the inferior endplate of the superior vertebral body and the isthmus, 1–2 cm from the spinous process. After the skin incision, put the obturator and working cannula on the lamina. Assembling the endoscopic system, laminoplasty, facetplasty, lateral recess decompression, ligamentum flavum resection, intervertebral disc and endplate preparation were performed. Make sure the decompression around the nerve root is complete and remove enough facet joints to allow the cage to pass through. The bone graft and cage insertion were in the same way above.

Subsequently, unlike the stand along procedure [19,20], the fused segments were fixed with bilateral percutaneous pedicle screws to achieve better intervertebral stability.

Instead of deliberate preoperative hemodilution [21], we supported patients by crystalloids during preoperative fasting according to physiological needs. Patients were treated with antibiotics intravenously for 24 h, and subcutaneous injection of low molecular weight heparin was used to prevent deep venous thrombosis from the first postoperative day. There was no drainage in this study. We recommend that ambulation training get started under the guidance of physiotherapists and protection of waistline from the first postoperative day and walk at least 200 m if the pain is bearable.

### 2.3. Calculation of Patient’s Blood Loss

The preoperative patient’s blood volume (PBV) was calculated by the Nadler [22] formula: PBV(L) = height (m)^3^ × K1 + weight (kg) × K2 + K3, in which K1 = 0.3669, K2 = 0.03219, K3 = 0.6041 for male and K1 = 0.3561, K2 = 0.03308, K3 = 0.1833 for female. The total blood loss (TBL) was calculated by the Gross [23] formula: TBL (L) = PBV × (Hct_pre_ − Hct_post_)/Hct_ave_, Hct_ave_ = (Hct_pre_+ Hct_post_)/2. In this study, the Intraoperative blood loss (IBL) during the operation was reckoned by infiltrated gauzes during the percutaneous pedicle screws fixation process and the estimation in the endoscopic process. Hidden blood loss: HBL = TBL − IBL.

### 2.4. Statistical Analysis

The data were analyzed using SPSS v26.0 for Mac (IBM Corp., Armonk, NY, USA). Continuous variables are expressed in terms of mean ±standard deviation (x ± s) or median (interquartile range) for normal data and non-normal data, respectively. Pearson’s correlation analysis (for normal data) and Spearman’s correlation analysis (for non-normal data) were performed to identify possible risk factors for HBL. The data met the criteria of multiple linear regression and it was conducted by stepwise option, including sex, age, BMI, hypertension, diabetes, ASA classification, fusion levels, surgical approach type (the count of trans-Kambin approach and interlaminar approach), surgery time, preoperative RBC, HGB, Hct, PT, INR, APTT, Fg, postoperative mean arterial pressure, postoperative heart rate, IBL, PBV to ascertain the factors. Factors with collinearity are eliminated.

## 3. Results

Three cases were excluded, including one case with a history of aspirin use and two cases with clopidogrel. A total of 96 patients (23 males and 73 females, age range: 30–85 years, 126 levels) were enrolled in this study. Of the 126 operative levels, 80 were fused via the trans-Kambin approach and 46 via the interlaminar approach. There were 68, 26, and two patients who underwent 1, 2 and 3 levels operation, respectively. All patients underwent the operation successfully, one patient had distributive shock and one patient had acute cerebral infarction after operation. These complications were effectively controlled after treatment. Thirty patients (eight males and 22 females) developed anemia. There were no other operative complications, such as poor incision healing. All patient demographics are presented in Table 1.

The total blood loss was 303.56 (120.49, 518.43) mL (median, (IQR)), of which hidden blood loss was 240.11 (65.51, 460.31) mL, accounting for 79.10% of TBL (Table 2).

The Pearson or Spearman correlation analysis demonstrated that following parameters was statistically significant: hypertension (*p* = 0.010), fusion levels (*p* = 0.000), trans-Kambin approach (*p* = 0.002), operative time (*p* = 0.001), IBL (*p* = 0.001) (Table 3).

Correlation coefficients of factors related to HBL are summarized in Table 4. Multiple linear regression analysis indicated that fusion levels (*p* = 0.002), age (*p* = 0.003), hypertension (*p* = 0.000), IBL (*p* = 0.012), PT (*p* = 0.016), preoperative HBG (*p* = 0.037) were the possible risk factors for HBL.

## 4. Discussion

The calculation of intraoperative blood loss is usually based on the amount of gauze infiltrated (or weighing), volume in suction canisters subtracting irrigation fluid, etc. Lotke P An et al. [24] found that there was a big difference between the calculated blood loss and the estimated intraoperative blood loss. Sehat et al. [10] described that invisible bleeding caused by postoperative blood infiltrated into the interspace of muscle, potential compartments and hemolysis led to this. The concept of “hidden blood loss” was proposed. Due to the existence of hidden blood loss, the total blood loss could be much more than the visible blood loss seen during the operation [25,26], which may lead to postoperative complications, such as anemia and poor incision healing condition [18,26]. It poses a threat to the patient’s perioperative safety and rehabilitation. As a result, hidden blood loss has received more attention by surgeons.

Hidden blood loss is calculated by Hct, and perioperative fluid infusion may lead to hemodilution and lower Hct. Even so, the calculation of hidden blood loss is different from hemodilution. The diagnosis of anemia based on hemoglobin is confounded by plasma volume, and excessive fluid may cause dilution anemia. Some studies have also taken advantage of this property: reducing Hct in different ways, so that even if the same volume of blood is lost during surgery, the loss of HGB and RBC will be less [21,27]. However, there is no related study on the effect of this practice on tissue oxygen supply in spinal surgery. On the other hand, the assessment of volume and body fluid balance is still difficult [28].The kidneys should eliminate the excessive fluid and there may be a fluid shift toward the extravascular space. Greenfield et al. [29] found that 20 min after rapid infusion of crystalloids, hemoglobin concentration returned to the baseline level. Gross formula [23] can correct hemodilution and calculate the amount of blood loss more accurately. It is not only because of the invisibility of the blood loss, but also because Gross formula corrects unpredictable fluid balance, such as hemodilution, making hidden blood loss more meaningful.

Even in minimally invasive surgery, hidden blood loss cannot be ignored. In previous studies, Zhou Y et al. [26] reported the total blood loss of MIS-TLIF reached 488.4 ± 294.0 mL, 52.5% of total blood loss (772.5 ± 328.8 mL). The hidden blood loss of OLIF reported by Zhu L et al. [18] was 809.0 ± 358.8 mL, accounting for 92.4% of the total blood loss (875.3 ± 391.4 mL). A study on XLIF blood loss [30] has similar results. In this study, the amount of hidden blood loss was 240.11 (65.51, 460.31) mL, accounting for 79.10% of the total blood loss, compared with the results of Ao, S. et al. [31] were similar (378.14 ± 139.05 mL). Less soft tissue invasion and more accurate bone resection minimized the range of injury. All of the above are beneficial to reduce hidden blood loss. However, it is unknown whether spontaneous hemolysis was associated.

The exact cause of hidden blood loss is still unclear, and current studies are exploring its possible risk factors. Hidden blood loss of MIS-TLIF may be related to the thickness of subcutaneous fat, and that in the OLIF procedure may be related to the length of the surgical approach. In addition, potential compartments, such as dead spaces left over after suture, may be an important influencing factor [32]. Although the risk factors of hidden blood loss are not really clear, one point could still be confirmed: the less invasive the operation is, the less the blood loss is. Compared with traditional open surgery, minimally invasive surgery has less blood loss, not only a smaller incision, less invasion of soft tissue, but also may reduce the possibility of potential compartments after closing the incision. As a recent emerging spinal surgery technology, Endo-LIF can meet the requirement of effective decompression and satisfactory fusion rate [33,34]. It is characterized by several incisions of about 1 cm, which greatly reduces the surgical trauma caused by paraspinal soft tissue dissection or traction and, unlike OLIF, avoids invading the superficial venous plexus and segmental arteries.

Spearman correlation analysis presented that hypertension, fusion levels, trans-Kambin approach, operative time, IBL are the possible factors of hidden blood loss. In the stepwise multiple linear regression model, fusion levels, younger age, hypertension, PT, preoperative HBG are selected as independent variables. This suggests that the indexes above may be related to hidden blood loss. There are some differences in the results between the two statistical methods, and the correlation of the former may be caused by confounding factors.

**Figure 1:**
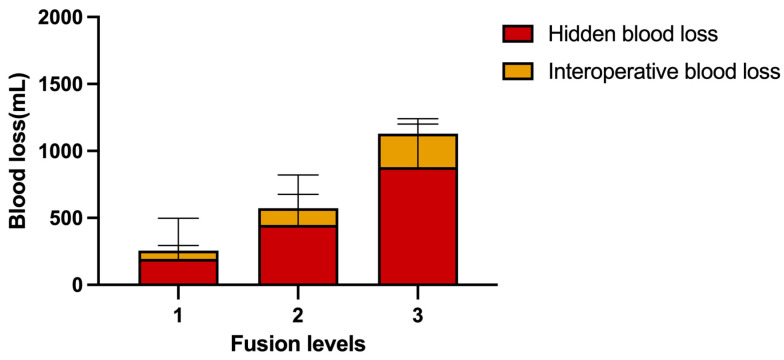
Mean blood loss between different fusion levels.

Fusion levels is an independent risk factor for HBL and its contribution to HBL is particularly significant. The increase of fusion levels doubles the invasion of bone and soft tissue, and more vascular injuries are possible. More cartilage endplate curettage also means more osseous endplate bleeding areas. As a result, blood loss in a multi-level procedure has increased significantly (Figure 1). Compared with some previous studies of open or minimally invasive spinal surgery [16,17,25,35], the increase in fusion levels is an important factor. In the correlation analysis, both the trans-Kambin approach and operation time were related to HBL. In multiple linear regression, the trans-Kambin approach and operation time are excluded. The trans-Kambin approach has a longer corridor length than the interlaminar approach, which seems to increase blood loss, but the difference between the channels created by blunt separation of soft tissue with an incision less than 1 cm is very slight. Most of the segments were fused via the trans-Kambin approach, especially in multi-level surgery. So, the correlation between the trans-Kambin approach and blood loss can be explained. The interlaminar approach was performed more commonly in the L5/S1 segment. It also represents the advantage of Endo-LIF, which is different from other minimally invasive surgery [26], the thickness of the soft tissue has no contribution to blood loss. The increase of fusion levels leads to multiple potential compartments and multiple bone and soft tissue injuries, which produces more oozing surfaces and more areas for blood to infiltrate to. At the same time, the increase of fusion levels also means that puncture, surgical corridor establishment, exposure, discectomy, endplate preparation, percutaneous pedicle screw fixation, and other operations will also be doubled. So, the operation time is likely to increase significantly. In other words, there may be no causal relationship between the increase of operation time and hidden blood loss, both of them are the results of the increase in fusion levels. The multiple linear regression model can correct the confounding factors in the correlation analysis. Finally, between fusion levels and operation time, only the latter was included as an independent risk factor. Therefore, we speculate that these two factors may be caused by more fusion levels.

In both statistical methods, hypertension has become an independent risk factor, which is similar to previous reports [36,37], as the blood pressure of patients must be controlled in preoperative preparation. Intraoperative blood pressure is relatively stable with the assistance of anesthesiologists, and the statistical results show that there is no significant correlation between postoperative mean arterial pressure and HBL. The mechanism of hypertension on HBL may be that muscle and other tissue is difficult to stanch spontaneously postoperatively due to the weakening of vascular elasticity, rather than the increase of pressure. Therefore, patients with hypertension and poor vascular conditions may have a more significant tendency of blood loss.

About the factor—age has a contradictory conclusion in different studies. Wang, H. et al. [35] believed that HBL is positively correlated with age; In the study of Lei, F. et al. [38], age is not related to the HBL of PLIF surgery; Shima, K. et al. [17] found that younger age is related to HBL, which is the same as the results of this study. These differences may be due to the different age distribution of patients reviewed. Yin, H. et al. [39] believed that blood may be more likely to infiltrate into the muscle interspace in elderly patients due to poorer vascular conditions and muscle wastage. On the other hand, more blood loss in young patients may related to their stronger paraspinal muscles with better blood supply, which may be more likely to lose blood. Additionally, older patients are more likely to be in a hypercoagulable state.

In stepwise multiple linear regression, prolonged PT and preoperative HBG were also associated with HBL. Previous studies [25,26,40] also suggested that preoperative blood routine examination and coagulation indexes were related to HBL. With a similar conclusion, Liu, X. et al. [41] considered that higher preoperative levels of HGB and Hct may be involved in the process of blood accumulation in the potential compartment. The risk factors analyzed on blood coagulation function were different among studies. The conclusion of Cai, T., et al. [40] was that hidden blood loss is positively correlated with INR, while the studies of Wang, H. et al. [35] show that there is a negative correlation between Fg and hidden blood loss. It is relatively easy to explain that a slight decrease in coagulation function within the normal range will increase the risk of HBL.

Although it is difficult to estimate the amount of bleeding due to irrigation during endoscopic surgery, IBL can still predict the amount of hidden blood loss after operation to a certain extent, but it is worth noting that this not only requires the operator to have rich experience in judging intraoperative bleeding. This is a relatively subjective parameter, and IBL only accounts for less than 20% of the total blood loss, which may lead the underestimation of actual amount of blood loss.

In short, from the results of this study, fusion levels, younger age, hypertension, PT, preoperative HBG are possible objective risk factors for hidden blood loss in Endo-LIF surgery. We hope to be able to predict the amount of hidden blood loss by these indicators and make better preparation and more timely intervention (such as more transfusion preparation, reservation of ICU, iron treatment, etc.) for patients who may have more hidden blood loss, while postoperative HGB levels are used as clinical criteria for treatment and transfusion [42].

In addition, the TBL of five patients in this study reached more than 1000 mL. Clinicians should pay more attention to the fact that significant blood loss may still occur even if full-endoscopic surgery was performed to avoid adverse outcomes. Of the 30 patients who developed anemia, 11 had HBG levels below 100 g/L, and they were five times more likely to develop surgical site infection than those without anemia (4.7%). Due to the relatively strict blood transfusion threshold limit, under the prerequisite of close observation of anemia symptoms, signs, and laboratory examination, to ensure that there is no further active bleeding in the surgical area, even if the HGB is in the range of 70–80 g/L, we do not carry out blood transfusion treatment for patients [42]. We intervened these patients with iron and Roxadustat, and there was no infection during the follow-up. Yang, Y. et al. [15] compared the blood loss of 21 patients with MIS-TLIF and 20 patients with open TLIF surgery. The total blood loss of minimally invasive surgery was less than that of open surgery (mean 355.3 vs. 538.6 mL). In the study of Ao, S. et al. [31], Endo-TLIF also had less blood loss than MIS-TLIF surgery (492.71 ± 150.19 vs. 698.11 ± 206.62 mL). This means the surgical trauma is closely related to the amount of blood loss. Although significant hemoglobin loss may occur after Endo-LIF, the minimally invasive ideas and pursuing the possibility of less injury are still worth implementing. However, the learning curve of Endo-LIF is relatively long, and a great deal of experience of spinal endoscopic surgery is required before primary fusion surgery.

There are some limitations to the present study that should be addressed. First of all, this is a single-center retrospective study with a small number of patients, which has the shortcomings of bias and relatively insufficient samples. In the second place, hidden blood loss in current studies was calculated by different time points of postoperative blood routine examination. In this study, we used the data of the first postoperative day to evaluate hidden blood loss. In addition to the methods used in this work, hidden blood loss calculated by second or third postoperative days and the lowest Hct value within 5 days postoperatively were also be used. This may lead to a lack of consistency among studies. The standalone lordotic endoscopic wedge lumbar interbody fusion (LEW-LIF) was not included in this study. Although a previous study [18] showed that combined posterior fixation contributed little to the HBL, further exploration was still needed in Endo-LIF surgery. In this study, we excluded the factors that may affect coagulation in order to more accurately analyze the effect of the Endo-LIF procedure itself on hidden blood loss. Including but not limited to these previous studies on hidden blood loss [15,18,26], cases with serious medical diseases that may affect coagulation, as well as the history of antiplatelet and anticoagulant drugs use are excluded. It could be for similar reasons. Although Foss, N.B. et al. [13] have suggested that these drugs may affect hidden blood loss in hip fractures, in a larger sample size of a minimally invasive elective spinal surgery study [43], this does not increase the blood loss and blood loss-related complications. These need to be further studied with a larger sample size in the future We have also considered indicators, such as postoperative HGB, HGB loss should be considered for risk factors of HBL. Further, we found that HGB and Hct have a very close relationship [44]. When HGB changes, Hct will change with it. Because hidden blood loss is calculated by Hct (the difference is that the hemodilution is corrected by Gross formula), there will be a strong correlation if indicators such as HGB loss are included in the regression equation. This interferes with other preoperative indicators that may be of reference value. Therefore, we referred to previous studies on HBL [13,18,25,45] which did not include postoperative HGB, HGB loss, and other postoperative indicators. These indicators need further research in statistics, mathematics, and other aspects.

## 5. Conclusions

The current study indicated that the Endo-LIF procedure is associated with a minimal amount of perioperative HBL among lumbar interbody fusion surgery. The fusion levels, younger age, hypertension, prolonged PT, preoperative HBG were the possible independent risk factors of HBL during Endo-LIF. Even though the blood loss caused by soft tissue invasion has been minimized, attention should also be paid to the invisible blood loss in Endo-LIF surgery, especially when multi-level surgery is required. The increase of surgical segments will lead to a considerable blood loss.

## Figures and Tables

**Table 1:** Statistical analysis of the demographic parameters.

Parameters	Statistics Median (IQR) or (x ± s)
Age(year)	59 (52, 69)
Height(cm)	158.83 ± 7.16
Weight(kg)	60.45 ± 9.21
BMI(kg/m^2^)	23.93 ± 3.21
Sex(n)	
Male	23
Female	73
Hypertension	30
Diabetes	10
ASA classification (I/II/III)	59/31/6
Surgical levels (1/2/3)	68/26/2
Surgical approach type (n)	
Trans-Kambin	80
Interlaminar	46
Operative time (min)	217.5 (175, 269.5)
Postoperative hospital stay (d)	5 (4, 7)
Preoperative PLT count (10^9^/L)	229.01 ± 54.53
Preoperative RBC (10^12^/L)	4.37 (4.04, 4.69)
Preoperative HGB (g/L)	129.55 ± 13.88
Preoperative Hct	0.389(0.367, 0.411)
PBV (mL)	3607 (3309, 4007)
PT (s)	10.61 ± 0.63
INR	0.881 ± 0.056
APTT (s)	24.1 (22.3, 26.3)
Fg (s)	2.82 ± 0.54
Postoperative mean arterial pressure (mmHg)	113.62 ± 8.53
Postoperative heart rate (n/min)	74.56 ± 7.13

ASA classification indicates American Society of Anesthesiologists Physical Status classification; PLT, platelets; RBC, red blood cell; HGB, hemoglobin; Hct, hematocrit; PBV, patient blood volume; PT, prothrombin time; INR, international normalized ratio; APTT, activated partial thromboplastin time; Fg, fibrinogen; BMI, body mass index; IQR, interquartile range.

**Table 2:** Perioperative blood loss in the patients.

Parameters Statistics	Median (IQR)
IBL	50 (50, 100)
TBL	303.56 (120.49, 518.43)
HBL	240.11 (65.51, 460.31)
Hidden blood loss in total (%)	79.10

IBL indicates Intraoperative blood loss. TBL, total blood loss. HBL, hidden blood loss.

**Table 3:** Results of the Pearson or Spearman correlation analysis for HBL.

Parameters	Sig (Two-Tailed)	*p*
Age	−0.150	0.144
BMI	−0.058	0.575
Sex	0.062	0.548
Hypertension	0.263	**0.010**
Diabetes	0.097	0.346
ASA classification	0.065	0.532
Fusion levels	0.394	**0.000**
Trans-Kambin approach	0.308	**0.002**
Interlaminar approach	−0.010	0.925
Operative time	0.329	**0.001**
Preoperative PLT count	0.035	0.735
Preoperative RBC	−0.038	0.717
Preoperative HGB	0.077	0.458
Preoperative Hct	0.118	0.252
PT (s)	0.130	0.208
INR	0.129	0.209
APTT (s)	−0.009	0.932
Fg (s)	−0.036	0.725
Postoperative mean arterial pressure	−0.011	0.916
PBV	−0.054	0.602
IBL	0.329	**0.001**

**Table 4:** Results of multivariate linear regression analysis for hidden blood loss.

**Coefficients**	**Unstandardized β**	**SE**	**Standardized β**	**t**	***p***
Fusion levels	227.127	70.442	0.330	3.224	0.002
Age	−7.862	2.607	−0.268	−3.016	0.003
Hypertension	244.873	67.629	0.325	3.621	0.000
IBL	1.214	0.471	0.254	2.577	0.012
PT	121.534	49.306	0.217	2.465	0.016
Preoperative HBG	4.615	2.179	0.183	2.118	0.037

## Data Availability

Not applicable.
